# RBM14 drives prostate cancer metastasis via stabilizing HK2 mRNA to activate glycolysis and H3K18 lactylation

**DOI:** 10.1038/s41420-026-03131-w

**Published:** 2026-04-30

**Authors:** Zhenhong Liu, Haixin Guo, Zhijiao You, Haichao Lin, Weihui Liu, Jiabi Chen, Qingliu He, Wei Zhuang

**Affiliations:** 1 Department of Urology, The Second Affiliated Hospital of Fujian Medical University, Quanzhou, 362000 Fujian China; 2 Department of Ultrasound, The Second Affiliated Hospital of Fujian Medical University, Quanzhou, 362000 Fujian China; 3 Department of Urology, Jinjiang Municipal Hospital. No. 16, Luoshan Section, Jinguang Road, Luoshan Street, Jinjiang, Quanzhou, Fujian China

**Keywords:** Prostate cancer, Cell migration

## Abstract

Metastasis is a leading cause of poor prognosis in prostate cancer (PCa), yet its underlying regulatory mechanisms remain incompletely understood. Following the establishment of highly invasive PC-3M cell lines, RBM14 expression was found to be significantly elevated in highly invasive cells. Furthermore, RBM14 was upregulated in PCa tissues and positively correlated with adverse clinicopathological features. Functional assays demonstrated that RBM14 significantly promoted PCa cell metastasis in vitro and in vivo. Mechanistically, RBM14 bound HK2 mRNA via its RRM1/2 domains to enhance HK2 stability, thereby upregulating HK2 expression. This increased HK2 level boosted PCa cells’ glycolytic capacity, which in turn led to increased global lactylation, especially in histone H3 lysine 18 lactylation (H3K18la). The elevated H3K18la preferentially enriched at the promoters of metastasis-related genes, further upregulating their expression. Importantly, combining RBM14 knockdown with 2-DG exerted a synergistic inhibitory effect on PCa metastasis. Collectively, this study identifies RBM14 as a key regulator of PCa metastasis via the HK2-glycolysis-H3K18la axis, providing a potential therapeutic target for combating PCa metastasis.

## Background

Prostate cancer (PCa) ranks among the most common malignancies and a leading cause of cancer-related death in men globally [1, 2]. Although early detection is increasingly routine, about 15% of patients are diagnosed with metastatic disease [3]. For patients with metastatic prostate cancer (mPCa), androgen deprivation therapy (ADT), novel hormonal therapy (NHT), docetaxel-based chemotherapy, and radiotherapy serve as first-line treatment modalities [4, 5]. While initially effective, these treatments often lead to acquired resistance, resulting in a median overall survival of less than three years [6]. Therefore, discovering new molecular drivers of PCa metastasis is essential for developing targeted therapies and improving clinical outcomes.

RNA-binding motif (RBM) proteins represent a major subclass of RNA-binding proteins (RBPs) that are critical for maintaining transcriptome integrity [7]. They interact with various RNA types, such as pre-mRNAs, mRNAs, and long non-coding RNAs (lncRNAs), to regulate multiple post-transcriptional events, including splicing, mRNA stability, nuclear export, and translation [8–10]. Growing evidence implicates RBM family members in cancer pathogenesis, where they can function as oncogenes or tumor suppressors [11–13]. For instance, RBM15 promotes laryngeal squamous cell carcinoma progression by modulating TMBIM6 stability [14]. Beyond their canonical RNA-binding roles, RBM14 also act as transcriptional co-factors or regulate telomere maintenance. Nevertheless, the function of RBM14 in prostate cancer remains largely unexplored.

Hexokinase 2 (HK2), the first rate-limiting enzyme of aerobic glycolysis, catalyzes glucose conversion to glucose-6-phosphate (G-6-P), a key step in initiating glycolysis [15, 16]. HK2 is commonly overexpressed in multiple cancers, including PCa, where its upregulation is associated with advanced disease stage, metastasis, and unfavorable prognosis [17]. Functional analyses demonstrate that HK2 facilitates tumor progression by augmenting glycolytic flux, promoting lactate production, and maintaining mitochondrial function [18]. Although prior research has largely focused on HK2 transcriptional control and post-translational modifications, the involvement of RNA-binding proteins in regulating HK2 expression remains inadequately explored.

In this study, by constructing a highly invasive PC-3M subline, we identified RBM14 was upregulated in PCa tissues and correlated with adverse clinicopathological parameters. We demonstrated that RBM14 enhances metastatic behavior both in vitro and in vivo. Mechanistically, RBM14 binds to HK2 mRNA through its RRM1/2 domains, increasing HK2 transcript stability and expression. Elevated HK2 levels augmented glycolytic activity in PCa cells, leading to increased global protein lactylation, particularly histone H3K18la. This rise in H3K18la enrichment at promoters of metastasis-related genes, further activated their transcription. Our results reveal a novel RBM14/HK2/glycolysis/H3K18la regulatory axis that promotes PCa metastasis, highlighting its potential as a therapeutic target for metastatic disease.

## Results

### Identification of RBM14 as a candidate PCa metastasis-related genes

**Fig. 1:**
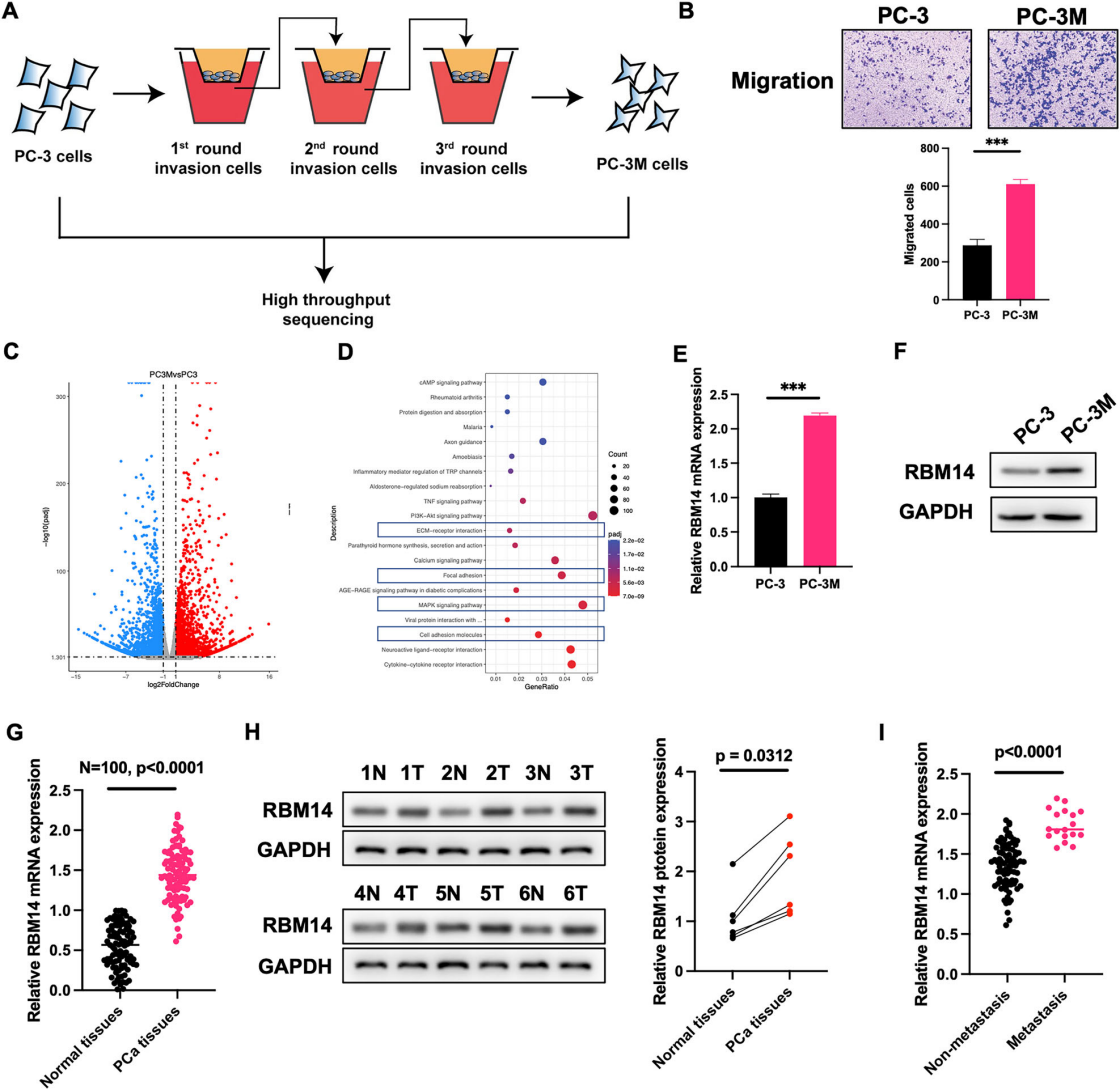
Identification of RBM14 as a candidate PCa metastasis-related genes. **A** Schematic diagram showing the construction of PC-3M cells via three round selections. **B** Transwell assay showing the migration ability of PC-3M and PC-3 cells. **C** Volcano diagram showing the differentially expressed genes in PC-3M and PC-3 cells. **D** KEGG pathway enrichment analysis showing the pathway DEGs enriched. **E** RT-qPCR assays showing the RBM14 mRNA expression in PC-3M and PC-3 cells. **F** Western blot assay showing the expression of RBM14 in PC-3M and PC-3 cells. **G** RT-qPCR assays showing the RBM14 mRNA expression in 100 paired PCa tissues. **H** Western blot assay showing the expression of RBM14 in 6 paired PCa tissues. **I** RT-qPCR assays showing the RBM14 mRNA expression in metastasis and non-metastasis patients. Data were presented at mean ±S.D. from at least three independent experiments.

To investigate genes involved in prostate cancer (PCa) metastasis, PC-3 cells were divided into two groups: one served as the Input group, and the other underwent three rounds of metastasis screening using Transwell chambers to obtain highly invasive PC-3 cells, which were designated as PC-3M cells (Fig. 1A). Compared with parental PC-3 cells, PC-3M cells exhibited significantly enhanced invasive capacity (Fig. 1B). Subsequently, RNA sequencing (RNA-seq) was performed on Input cells and PC-3M cells, revealing more than 5500 differentially expressed genes with the criteria of Log₂FC > 1 and *p* value < 0.05 (Fig. 1C). Meanwhile, KEGG pathway enrichment analysis showed that these DEGs were enriched in the “Focal adhesion”, “Cell adhesion molecules”, and “ECM-receptor interaction” pathway (Fig. 1D), which associated with tumor metastasis. Among these DEGs, RBM14, a member of the RBM family, was found to be significantly upregulated in PC-3M cells, prompting us to select RBM14 as the focus of subsequent investigations. Given that our research group has long focused on RBM family proteins, we have previously demonstrated that RBM47 inhibits renal cell carcinoma progression [19]; additionally, RBM19 promotes docetaxel resistance in PCa by regulating the lncRNA SNHG21, which protects PIM1 protein from ubiquitin-dependent degradation [20]. RBM14 was confirmed to be highly expressed in PC-3M cells via qRT-PCR and Western blot (WB) assays (Fig. 1E, F). Furthermore, RBM14 expression was found to be elevated in PCa patients through analysis of TCGA-PRAD database data (Supplementary Fig. S1A). Moreover, qPCR detection of 100 pairs of PCa tissues and adjacent normal tissues showed that RBM14 was highly expressed in PCa tissues (Fig. 1G), and WB assays further confirmed the upregulation of RBM14 in PCa tissues (Fig. 1H). Additionally, both TCGA-PRAD database and our PCa cohort showed that RBM14 expression was higher in patients with high Gleason scores (Supplementary Fig. S1B, C) and in patients with metastatic disease (Fig. 1I, Supplementary Fig. S1D). Finally, high RBM14 expression was found to be associated with poor disease-free survival in PCa patients (Supplementary Fig. S1F). Collectively, these results indicate that RBM14 is highly expressed in PC-3M cells and its expression correlates with the clinical characteristics of PCa patients.

### RBM14 promotes prostate cancer metastasis in vitro and in vivo

**Fig. 2:**
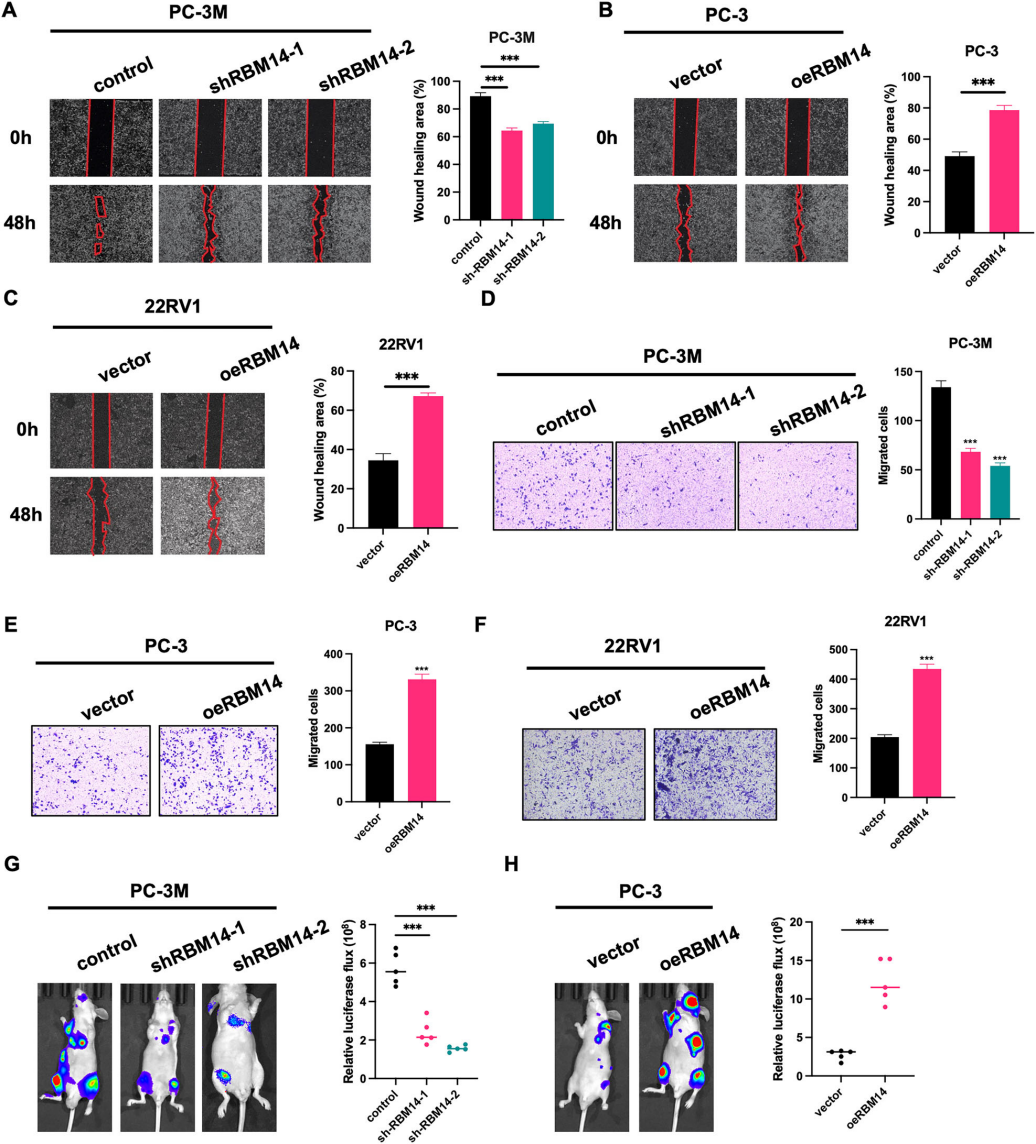
RBM14 promotes prostate cancer metastasis in vitro and in vivo. **A** Wound healing assays showing the migration ability of PC-3M cells with RBM14 knockdown or not. **B** Wound healing assays showing the migration ability of PC-3 cells with RBM14 overexpression or not. **C** Wound healing assays showing the migration ability of 22RV1 cells with RBM14 overexpression or not. **D** Transwell assays showing the migration ability of PC-3M cells with RBM14 knockdown or not. **E** Transwell assays showing the migration ability of PC-3 cells with RBM14 overexpression or not. **F** Transwell assays showing the migration ability of 22RV1 cells with RBM14 overexpression or not. **G** Mice intracardiac injection metastasis model showing the metastasis ability of PC-3M cells with RBM14 knockdown or not. **H** Mice intracardiac injection metastasis model showing the metastasis ability of PC-3 cells with RBM14 overexpression or not. Data were presented at mean ±S.D. from at least three independent experiments. ***p < 0.001.

To explore the role of RBM14 in PCa metastasis, we first knocked down RBM14 in PC-3M cells (which endogenously high-express RBM14) and overexpressed RBM14 in PC-3 and 22RV1 cells (which low-express RBM14). The efficiency of RBM14 knockdown and overexpression was verified (Supplementary Fig. S2A–D). Subsequent wound healing assays demonstrated that knockdown of RBM14 significantly reduced the migratory capacity of PC-3M cells (Fig. 2A), while overexpression of RBM14 enhanced the migration of PC-3 and 22RV1 cells (Fig. 2B, C). Furthermore, Transwell migration assays confirmed that RBM14 knockdown decreased the metastatic potential of PC-3M cells (Fig. 2D), whereas RBM14 overexpression promoted the metastasis of PC-3 and 22RV1 cells (Fig. 2E, F). Finally, we established a PCa metastasis mouse model by intracardiac injection of luciferase-labeled PC-3 cells with stable RBM14 overexpression. The results showed that overexpression of RBM14 significantly enhanced the metastatic capacity of PC-3 cells (Fig. 2G, H). Collectively, these data confirm that RBM14 promotes prostate cancer metastasis.

### RBM14 upregulates HK2 expression by directly binding to HK2 mRNA and enhancing its stability

**Fig. 3:**
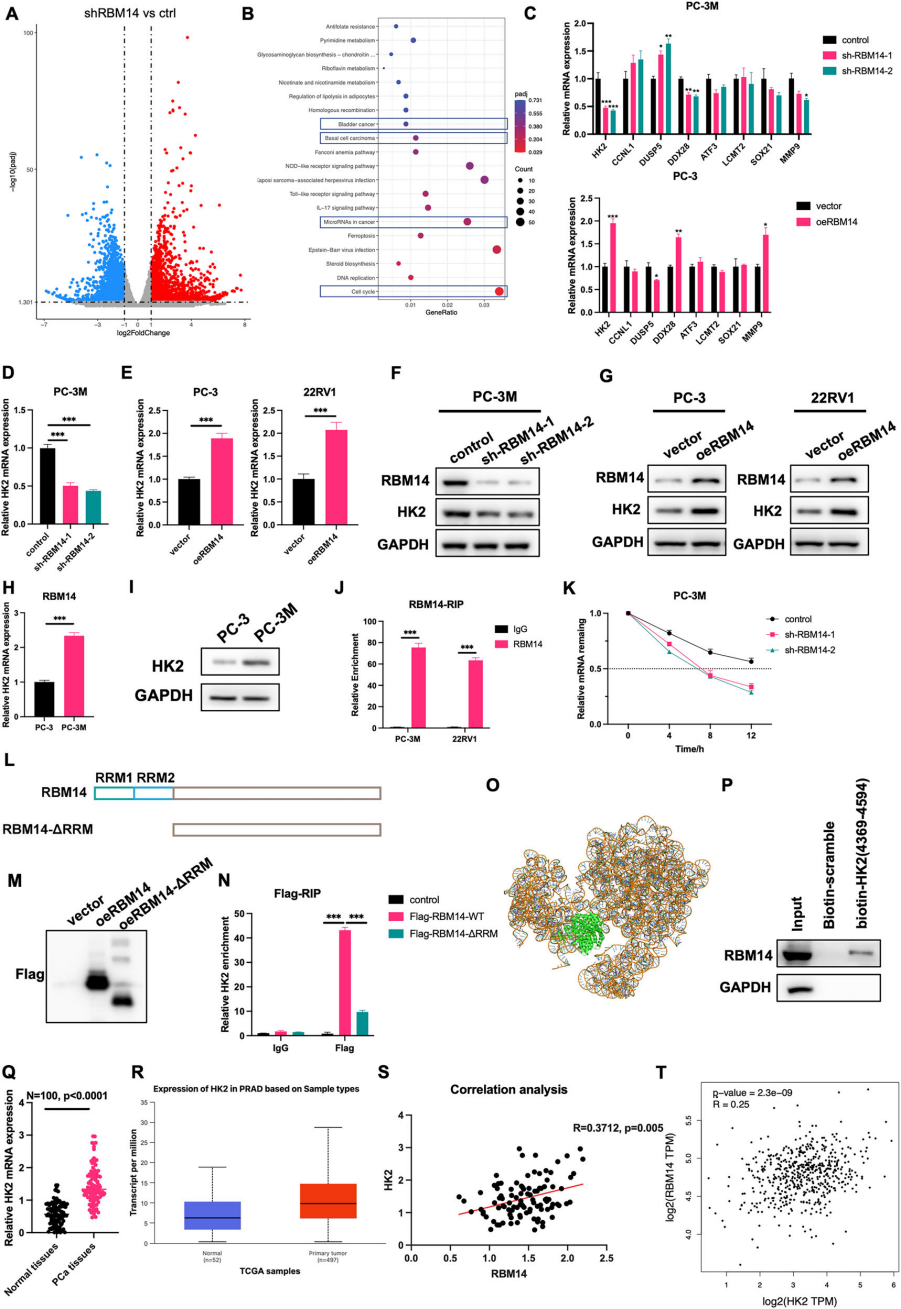
RBM14 upregulates HK2 expression by directly binding to HK2 mRNA and enhancing its stability. **A** Volcano diagram showing the differentially expressed genes in PC-3M cells with RBM14 knockdown or not. **B** KEGG pathway enrichment analysis showing the pathway DEGs enriched. **C** RT-qPCR assays showing the mRNA expression in PCa cells with RBM14 knockdown or overexpression. **D** RT-qPCR assays showing the HK2 mRNA expression in PC-3M cells with RBM14 knockdown or not. **E** RT-qPCR assays showing the HK2 mRNA expression in PCa cells with RBM14 overexpression or not. **F** Western blot assay showing the expression of HK2 in in PC-3M cells with RBM14 knockdown or not. **G** Western blot assay showing the expression of HK2 in in PCa cells with RBM14 overexpression or not. **H** RT-qPCR showing the expression of HK2 in PC-3M and PC-3 cells. **I** Western blot assay showing the expression of HK2 in PC-3M and PC-3 cells. **J** RIP assay showing the binding ability of RBM14 and HK2 mRNA. **K** RNA stability assay showing the HK2 mRNA stability in PC-3M cells with RBM14 knockdown or not. **L** The diagram illustrating the truncations of RBM14 proteins. **M** Western blot assay showing the expression of RBM14 and its trunction in PCa cells. **N** RIP-qPCR assay showing the binding capacity between HK2 and Full length or truncations of RBM14. **O** The docking model of HK2 and RBM14 predicted by Alphafold2 software. **P** RNA pulldown assay showing the binding capacity between RBM14 and the segement of HK2 mRNA (4369–4594 nt). **Q** RT-qPCR assays showing the HK2 mRNA expression in 100 paired PCa tissues. **R** HK2 mRNA expression was analyzed in TCGA-PRAD database. **S** Correlation analysis showing the positive correlation between SNHG21 and RBM19 in 100 paired PCa tissues. **T** TCGA-PRAD database analysis showing the positively correlation between HK2 and RBM14. Data were presented at mean ±S.D. from at least three independent experiments. *p < 0.05, **p < 0.01, ***p < 0.001.

To elucidate the mechanism by which RBM14 regulates PCa metastasis, we first performed RNA-seq on PC-3 cells with RBM14 overexpression, identifying more than 4100 differentially expressed genes (Log₂FC > 1, *p* value < 0.05) (Fig. 3A). Meanwhile, KEGG pathway enrichment analysis showed that these DEGs were enriched in the cancer-related pathways (Fig. 3B). Subsequently, we selected 8 genes associated with PCa progression from these differentially expressed genes as candidate targets for further investigation. qRT-PCR assays demonstrated that RBM14 regulated the expression of genes including HK2, DUSP5, and DDX28, with the most prominent regulatory effect observed on HK2 (Fig. 3C); therefore, HK2 was selected as the downstream target gene of RBM14. Next, validation via RBM14 overexpression and knockdown in PCa cells showed that RBM14 positively regulated HK2 expression at both the mRNA and protein levels (Fig. 3D–G). Moreover, HK2 was significantly highly expressed in PC-3M cells (Figs. 3H, I). Previous studies have reported that RBM family proteins are involved in RNA processing, including pre-mRNA splicing and mRNA stability regulation [7]. Therefore, we first examined whether RBM14 could regulate HK2 expression by directly binding to HK2 mRNA. RNA Immunoprecipitation (RIP) assays revealed that RBM14 could directly bind to HK2 mRNA (Fig. 3J). Furthermore, we found that knockdown of RBM14 reduced the stability of HK2 mRNA (Fig. 3K), while overexpression of RBM14 increased its stability (Fig. S3A). To clarify which domain of RBM14 mediates its interaction with HK2 mRNA, we constructed Flag-tagged RBM14 truncations with deletions of the RRM1 and RRM2 domains (Fig. 3L), and WB assays confirmed the successful construction of the Flag-tagged RBM14 truncation (Fig. 3M). RNA-IP assays showed that wild-type RBM14 could directly bind to HK2 mRNA, whereas the binding ability of the RBM14-ΔRRM mutant to HK2 mRNA was significantly reduced (Fig. 3N), suggesting that RBM14 recognizes and binds to HK2 mRNA via its RRM1 and RRM2 domains. Additionally, Alpha Fold2 prediction indicated that RBM14 mainly binds to the 4000–6000 bp region of HK2 mRNA (https://alphafoldserver.com/) (Fig. 3O). Further analysis using the catRAPID database predicted that the 4369–4594 bp segment of HK2 mRNA is the key site mediating the interaction between RBM14 and HK2 mRNA (http://service.tartaglialab.com/new_submission/catrapid_omicsv2_custom). We therefore designed biotin-labeled HK2 (4369–4594) probes and control probes for RNA pulldown assays, and the results confirmed that the 4369–4594 bp segment of HK2 mRNA is the critical site for RBM14-HK2 mRNA binding (Fig. 3P). Subsequently, HK2 was shown to be highly expressed in PCa tissues via detection in 100 pairs of PCa tissues and their adjacent non-tumor tissues (Fig. 3Q). Moreover, HK2 expression was found to be elevated in PCa patients through analysis of TCGA-PRAD database data (Fig. 3R). Further correlation analysis showed a positive correlation between HK2 and RBM14 mRNA levels in both the TCGA-PRAD database and our PCa cohort (Fig. 3S, T). Collectively, these results suggest that RBM14 directly binds to HK2 mRNA, enhances its stability, and ultimately upregulates HK2 expression.

### RBM14 enhances aerobic glycolysis in PCa cells

**Fig. 4:**
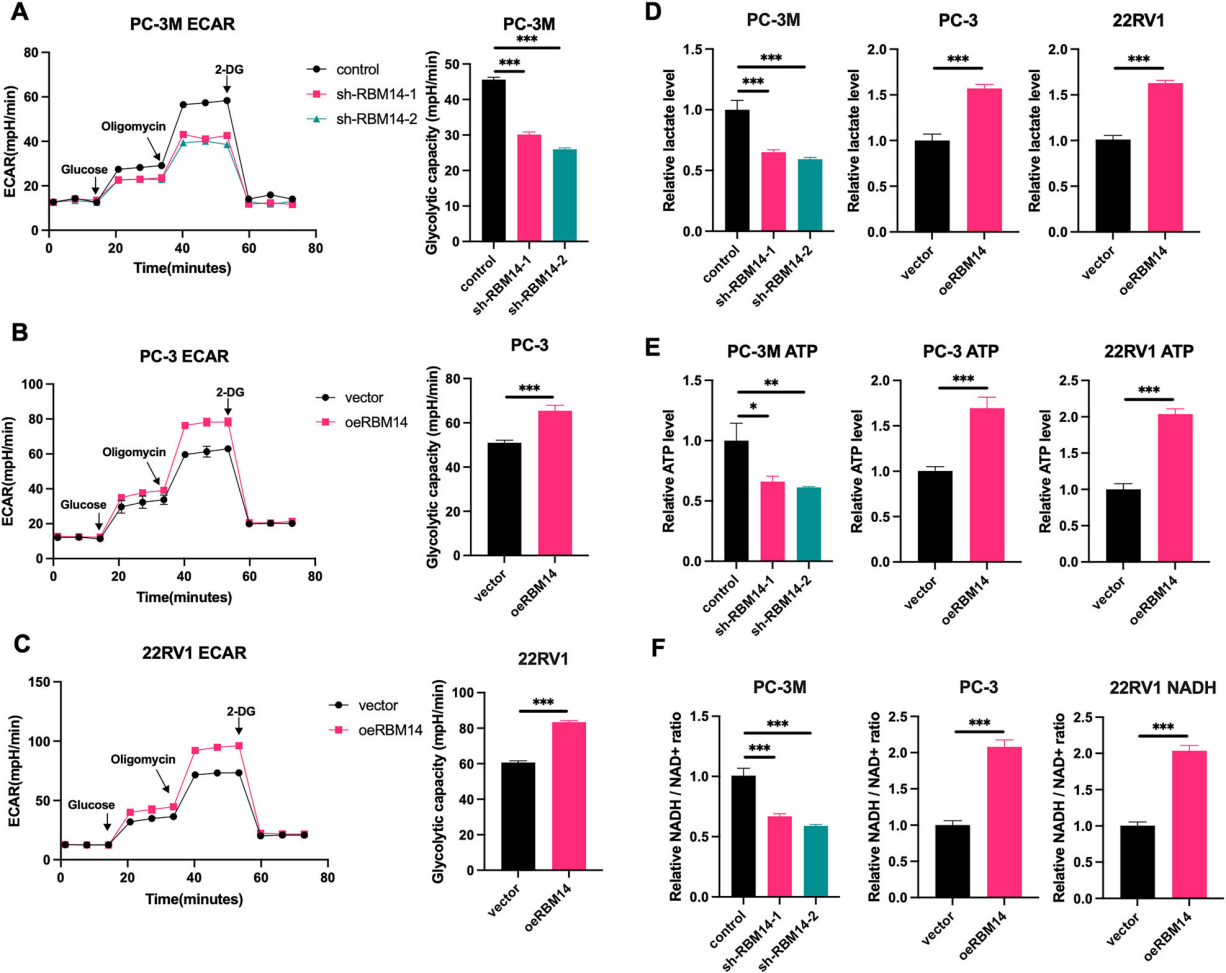
RBM14 enhances aerobic glycolysis in PCa cells. **A** ECAR assay showing the glycolytic capacity of PC-3M cells with RBM14 knockdown or not. **B** ECAR assay showing the glycolytic capacity of PC-3 cells with RBM14 overexpression or not. **C** ECAR assay showing the glycolytic capacity of 22RV1 cells with RBM14 overexpression or not. **D** The relative lactate production level in PCa cells with RBM14 knockdown or overexpression. **E** The relative ATP level in PCa cells with RBM14 knockdown or overexpression. **F** The relative NADH/NAD+ level in PCa cells with RBM14 knockdown or overexpression. Data were presented at mean ±S.D. from at least three independent experiments. ***p < 0.001.

HK2 is the first rate-limiting enzyme in aerobic glycolysis, which catalyzes the conversion of glucose to glucose-6-phosphate and is widely recognized to directly participate in the metabolic reprogramming of tumor cells [16, 17]. Given that RBM14 can regulate HK2 expression, we further investigated whether RBM14 modulates aerobic glycolysis in PCa cells. Extracellular Acidification Rate (ECAR) assays showed that knockdown of RBM14 significantly reduced the glycolytic capacity of PC-3M cells (Fig. 4A), whereas overexpression of RBM14 markedly increased the glycolytic capacity of PCa cells (Fig. 4B, C). Subsequent energy metabolism analysis revealed that RBM14 knockdown significantly decreased lactate production, ATP generation, and NADH/NAD+ ratio, while RBM14 overexpression exerted the opposite effects (Fig. 4D–F). Collectively, these results indicate that RBM14 promotes aerobic glycolysis in PCa cells, leading to increased lactate production and energy generation.

### RBM14 regulates PCa cell metastasis and glycolysis dependent on HK2

**Fig. 5:**
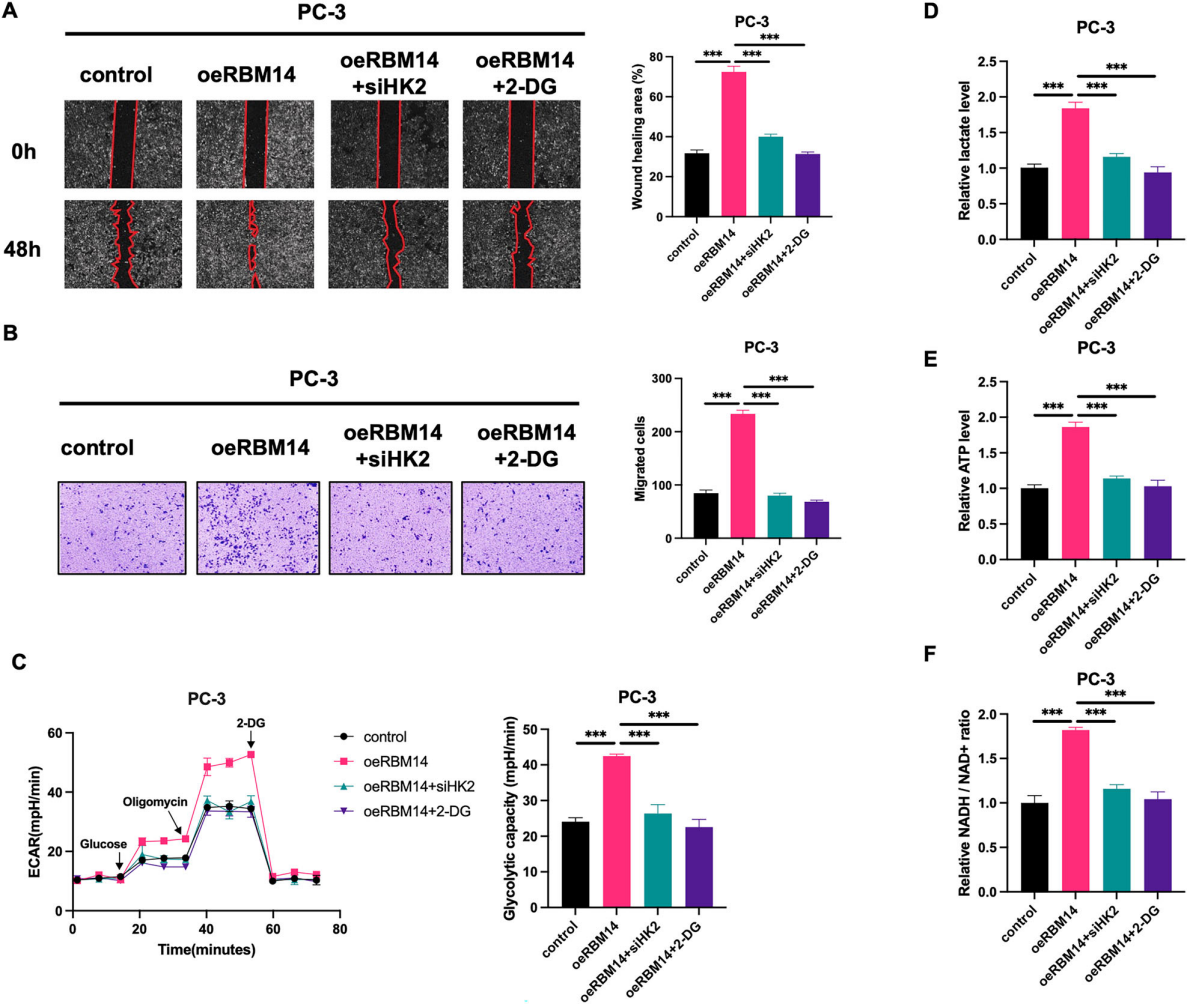
RBM14 regulates PCa cell metastasis and glycolysis dependent on HK2. **A** Wound healing assay showing the migration ability of RBM14 overexpression PC-3 cells with HK2 knockdown or added with 2-DG. **B** Transwell assay showing the migration ability of RBM14 overexpression PC-3 cells with HK2 knockdown or added with 2-DG. **C** ECAR assay showing the glycolytic capacity of RBM14 overexpression PC-3 cells with HK2 knockdown or added with 2-DG. **D** The relative lactate production level in RBM14 overexpression PC-3 cells with HK2 knockdown or added with 2-DG. **E** The relative ATP level in RBM14 overexpression PC-3 cells with HK2 knockdown or added with 2-DG. **F** The relative NADH/NAD+ level in RBM14 overexpression PC-3 cells with HK2 knockdown or added with 2-DG. Data were presented at mean ±S.D. from at least three independent experiments. ***p < 0.001.

To confirm whether RBM14 mediates PCa metastasis through HK2, we performed rescue experiments using HK2 siRNA and 2-DG, a pharmacological inhibitor of hexokinase activity. Wound healing and Transwell assays demonstrated that RBM14 overexpression enhanced PCa cell metastatic potential, an effect that was effectively reversed by specific knockdown of HK2. Notably, pharmacological intervention with 2-DG yielded a similar rescue effect (Figs. 5A, B), suggesting that the pro-metastatic role of RBM14 is linked to hexokinase-mediated glycolysis. Furthermore, ECAR assays showed that HK2 knockdown or 2-DG treatment reversed the RBM14 overexpression-induced increase in glycolytic capacity of PCa cells (Fig. 5C). Consistent with this, energy metabolism analysis revealed that HK2 knockdown or 2-DG treatment also abrogated the RBM14 overexpression-mediated elevation in lactate secretion, ATP production, and NADH/NAD+ ratio in PCa cells (Fig. 5D–F). Collectively, these results provide evidence that the RBM14-regulated metastatic phenotype is at least partially dependent on the HK2-driven glycolytic pathway.

### RBM14 upregulates H3K18 lactylation and metastasis-related gene expression via HK2

**Fig. 6:**
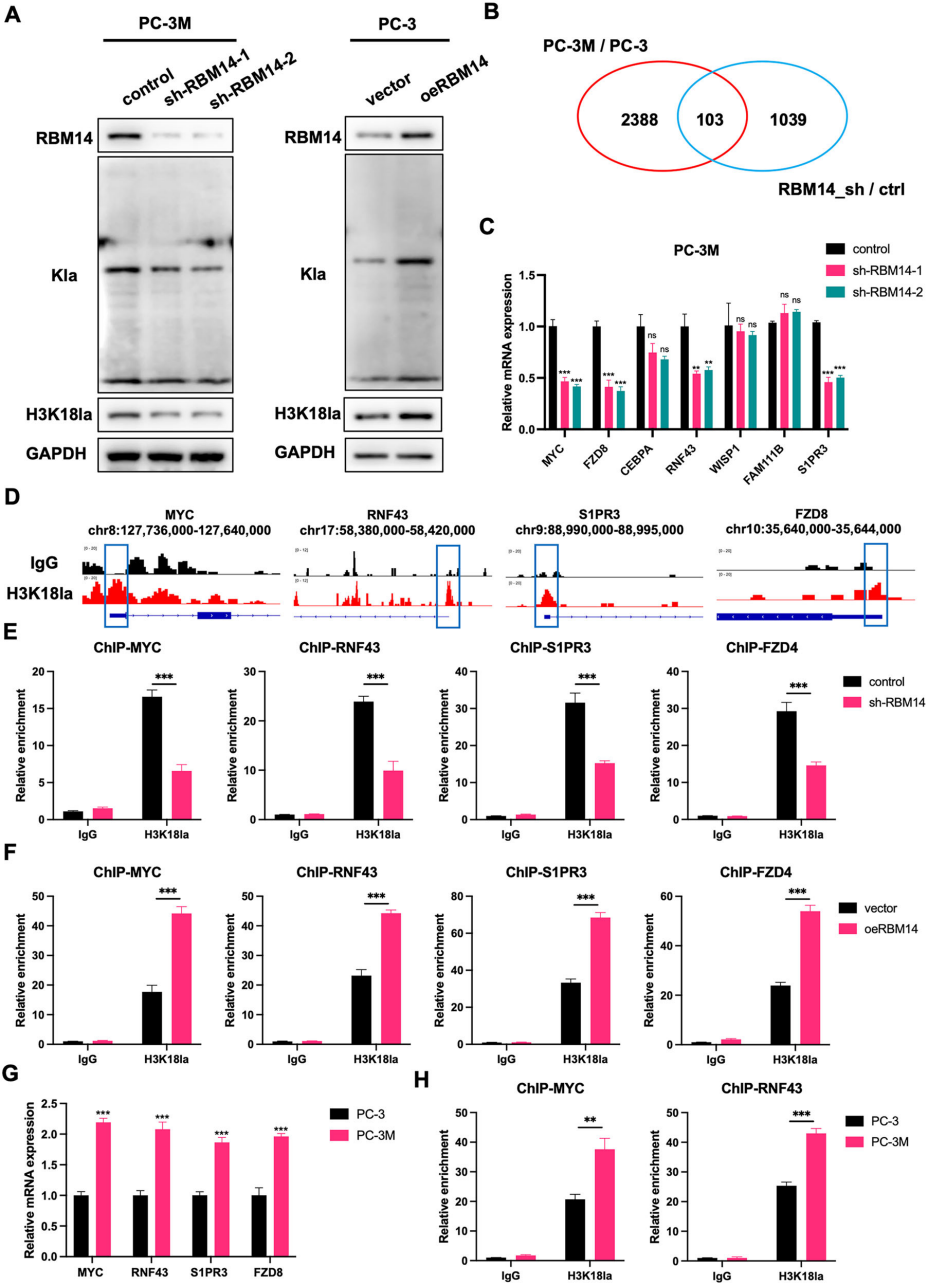
RBM14 upregulates H3K18 lactylation and metastasis-related gene expression via HK2. **A** Western blot assays showing the total lactylation and H3K18la modification level in PCa cells with RBM14 knockdown or overexpression. **B** Venn diagram showing the common DEGs in RNA sequencing data. **C** RT-qPCR showing the expression of MYC, RNF43, SIPR3, FZD8, CEBPA, WISP1, and FAM111B in PC-3M cells with RBM14 knockdown or not. **D** IGV diagrams illustrate the H3K18la peak in the promoter region of MYC, RNF43, SIPR3, FZD8 genes. **E** ChIP-qPCR assays showing the relative enrichment of H3K18la modification in the promoter region of MYC, RNF43, SIPR3, FZD8 genes with RBM14 knockdown or not. **F** ChIP-qPCR assays showing the relative enrichment of H3K18la modification in the promoter region of MYC, RNF43, SIPR3, FZD8 genes with RBM14 overexpression or not. **G** RT-qPCR showing the expression of MYC, RNF43, SIPR3, FZD8 in PC-3M and PC-3 cells. **H** ChIP-qPCR assays showing the relative enrichment of H3K18la modification in the promoter region of MYC, RNF43 genes. Data were presented at mean ±S.D. from at least three independent experiments. ns, not significant, **p < 0.01, ***p < 0.001.

Lactylation is a recently identified post-translational modification that was initially linked to the regulation of gene transcription through epigenetic mechanisms [21]. The primary driver of lactylation is lactate, a byproduct of the Warburg effect [22]. Our previous data indicated that RBM14 activates the glycolytic pathway by regulating HK2 expression, ultimately promoting lactate production. Thus, we explored whether RBM14 modulates lactylation in PCa cells. Western blot assays showed that RBM14 knockdown decreased the global lactylation level in cells, while RBM14 overexpression increased it (Fig. 6A). Notably, the lactylation level of proteins with a molecular weight of ~15 kDa (a size typically corresponding to histone lactylation) was prominently altered. Among histone lactylation sites, histone H3 lysine 18 lactylation (H3K18la) has been the most extensively studied [23, 24]. We therefore detected H3K18la levels and found a positive correlation between H3K18la modification and RBM14 protein expression (Fig. 6A), indicating that RBM14 regulates H3K18la modification. By integrating our transcriptome data (Log₂FC > 2, *p* value < 0.01), we prioritized MYC, RNF43, SIPR3, FZD8, CEBPA, WISP1, and FAM111B as representative pro-metastatic drivers for subsequent validation, given their established roles in cancer metastasis. (Fig. 6B). RT-qPCR assays revealed that RBM14 positively regulated MYC, RNF43, SIPR3, and FZD8 expression (Fig. 6C, Supplementary Fig. S4A). Furthermore, to identify the specific lactylation site in these genes’ promoter, the H3K18la ChIP-seq data was analyzed (GSE307050) (Fig. 6D). H3K18la ChIP-qPCR assays showed that RBM14 overexpression significantly increased H3K18la modification at the promoter regions of the MYC, RNF43, SIPR3, and FZD8, while RBM14 knockdown decreased H3K18la modification level (Fig. 6D–F). Additionally, MYC, RNF43, SIPR3, and FZD8 were confirmed upregulated in PC-3M cells compared with PC-3 cells (Fig. 6G), as well as the H3K18la modification level in their promotor (Fig. 6H, Supplementary Fig. S4B). These results suggest that RBM14 promotes the expression of metastasis-related genes by enhancing H3K18la modification at their promoter regions.

### Combination of RBM14 knockdown and 2-DG exerts a synergistic inhibitory effect on PCa cell metastasis

**Fig. 7:**
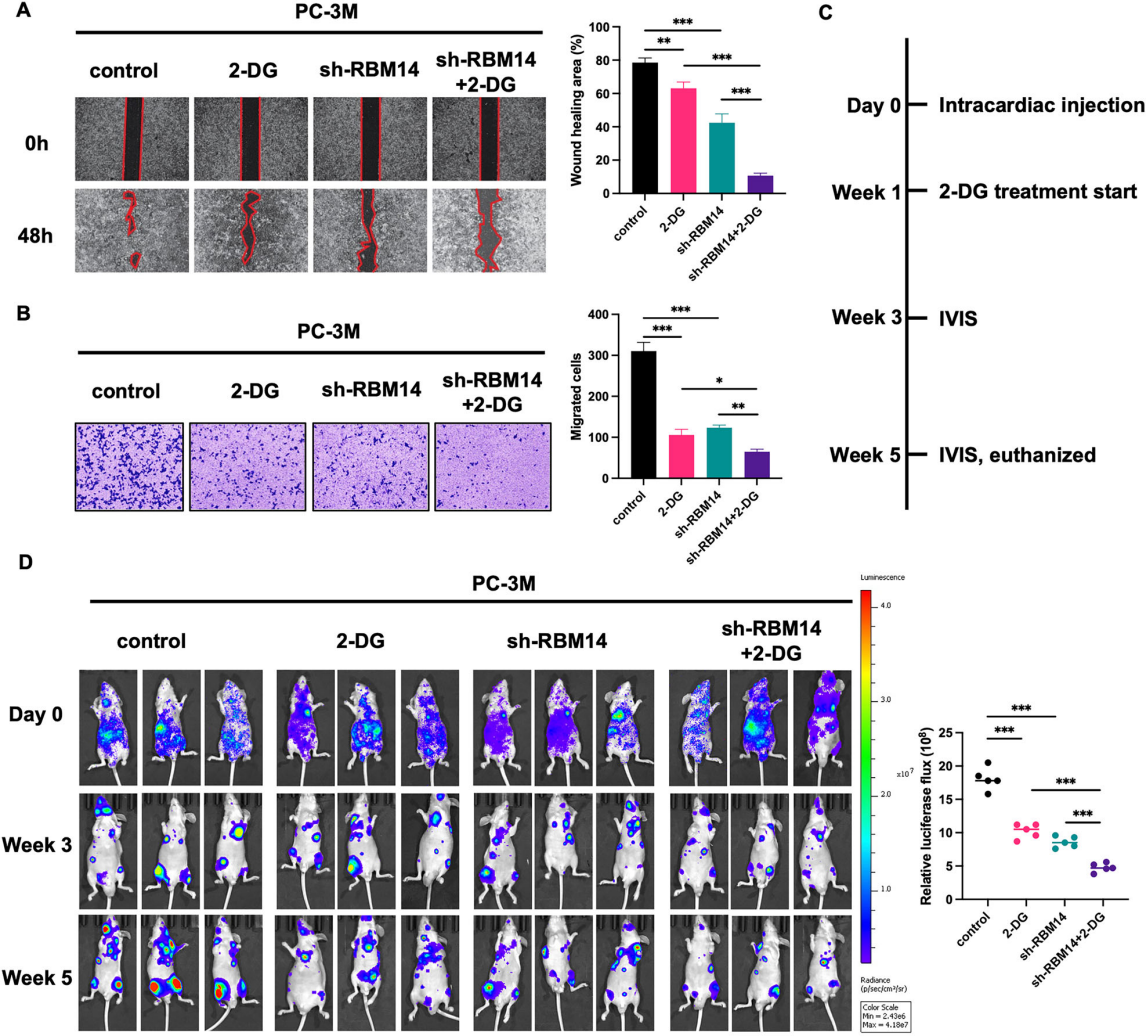
Combination of RBM14 knockdown and 2-DG exerts a synergistic inhibitory effect on PCa cell metastasis. **A** Wound healing assay showing the migration ability of PC-3M cells with 2-DG treatment and (or) RBM14 knockdown. **B** Transwell assay showing the migration ability of PC-3M cells with 2-DG treatment and (or) RBM14 knockdown. **C** Schematic diagram showing the time line of animal model. **D** Mice intracardiac injection metastasis model showing the metastasis ability of PC-3 cells with with 2-DG treatment and (or) RBM14 knockdown. Data were presented at mean±S.D. from at least three independent experiments. *p < 0.05, **p < 0.01, ***p < 0.001.

RBM14 knockdown inhibits PCa metastasis by reducing HK2 expression, whereas 2-DG blocks the first step of glycolysis by competitively inhibiting hexokinase, thereby disrupting cellular energy metabolism [25, 26]. Given their distinct mechanisms of regulating HK2-related glycolysis, we hypothesized that the combination of RBM14 knockdown and 2-DG would exert a synergistic inhibitory effect on PCa metastasis. Wound healing and transwell assays showed that both RBM14 knockdown and 2-DG alone effectively inhibited tumor metastasis, and their combination exhibited a synergistic inhibitory effect (Fig. 7A, B). Consistently, PCa metastasis mouse model by intracardiac injection demonstrated that RBM14 knockdown or 2-DG alone suppressed PCa cell metastasis, while their combination significantly enhanced this inhibitory effect on PCa cell metastasis (Fig. 7C, D). Collectively, these results indicate that the combination of RBM14 knockdown and 2-DG exerts a potent synergistic inhibitory effect on PCa cell metastasis.

## Discussion

Metastatic progression remains the principal factor limiting survival in prostate cancer (PCa) patients, underscoring the need to elucidate the molecular networks that drive metastasis for the development of effective therapies [27–29]. In this study, we established a highly invasive PC-3M subline through three rounds metastasis screening and identified RBM14 as a metastasis-associated gene via RNA sequencing. RBM14 expression was significantly upregulated in PCa tissues and correlated with high Gleason scores, lymph node or bone metastasis, and reduced disease-free survival, supporting its potential role as a prognostic biomarker.

RNA-binding motif (RBM) proteins play key roles in maintaining transcriptome integrity by regulating post-transcriptional processes such as splicing, mRNA stability, nuclear export, and translation [30, 31]. Multiple RBM family members have been implicated in tumorigenesis [32–34]. For example, RBM25 promotes colon cancer growth by modulating MNK2 alternative splicing [35], and RBM17 facilitates hepatocellular carcinoma progression through alternative splicing of CSAD pre-mRNA [36]. Nevertheless, the function of RBM14 in PCa metastasis remains largely unexplored. Here, using wound healing, transwell assays, and an intracardiac metastasis model, we demonstrated that RBM14 enhances the metastatic potential of PCa cells, identifying it as a driver of metastasis. Mechanistically, RBM14 binds to HK2 mRNA via its RRM1/2 domains, increasing HK2 transcript stability and expression. As a key rate-limiting enzyme in the Warburg effect, HK2 promotes tumor progression by enhancing glycolytic flux, lactate production, and mitochondrial function. Our data indicate that RBM14 modulates glycolytic capacity, lactate output, and ATP generation through HK2 upregulation, positioning RBM14 as a candidate upstream regulator of HK2-driven glycolysis in PCa.

Lactate, the end product of aerobic glycolysis, serves as a substrate for lactylation modifications [37]. Elevated lactate levels in cancer cells have been linked to increased H3K18la, which activates genes involved in proliferation and metastasis [38]. However, the upstream mechanisms linking glycolysis to H3K18la in PCa remain unclear. In this study, we found that RBM14 enhances lactate production via HK2 upregulation, leading to elevated global lactylation, particularly at H3K18. ChIP-qPCR analysis confirmed enrichment of H3K18la at promoters of metastasis-related genes (e.g., MYC, RNF43, SIPR3, and FZD8), promoting their expression. This finding establishes a RBM14/HK2/glycolysis/lactate/H3K18la/metastasis-related genes axis, which contribute to PCa metastasis.

A critical question arises as to how a global increase in intracellular lactate, driven by the RBM14-HK2 axis, achieves specific regulation of metastasis-related genes. Our findings suggest that this specificity is likely governed by differential chromatin accessibility. In cancer cells, the promoters of key oncogenes like MYC and RNF43 are typically maintained in an open chromatin configuration, making them more sensitive to fluctuations in metabolic cofactors compared to silenced genomic regions [39, 40]. While RBM14-mediated glycolysis promotes a broad increase in H3K18la, these ‘primed’ loci exhibit preferential enrichment and functional response. Indeed, our transcriptomic profiling revealed a wide array of over 1000 responsive genes, from which we validated a subset of critical metastatic drivers, emphasizing that RBM14 exerts a broad yet context-dependent influence on the epigenetic landscape.

2-DG is a competitive inhibitor of hexokinases, which showing efficacy in inhibiting glycolysis in multiple malignancies [25]. In our study, we found RBM14 targets HK2 at the post-transcriptional level. Considering that they target HK2 in different mechanism, we wonder whether combining RBM14 knockdown with 2-DG exerts a stronger effect in treating PCa. Both the in vivo and in vitro assays showing that combining 2-DG with RBM14-KD therapies showing promising synergistic inhibitory effect on PCa cell metastasis.

Despite the novel insights provided by the RBM14-HK2-H3K18la axis in prostate cancer metastasis, several limitations should be acknowledged. First, our initial screening and functional validations were primarily conducted using established PCa cell models. While these models are instrumental in mechanistic studies, they may not fully recapitulate the complex microenvironment of metastatic niches in vivo. Future research utilizing in vivo selection would further substantiate the essential role of the RBM14/glycolysis axis in late-stage disease progression. Second, due to clinical and ethical constraints, our analysis of human specimens was limited to primary tumor and paracancerous tissues. Since patients with advanced metastasis often lack surgical indications for the resection of metastatic lesions, direct validation of the RBM14-H3K18la signature in human metastatic foci was not feasible. Finally, although we identified specific downstream targets of RBM14 via RNA-seq and ChIP-qPCR, a global H3K18la ChIP-seq profile was not obtained in the current study. A more comprehensive genomic analysis in future research will provide a holistic view of the epigenetic landscape regulated by RBM14, potentially uncovering additional oncogenic drivers sensitive to lactate-mediated lactylation.

## Conclusion

**Fig. 8:**
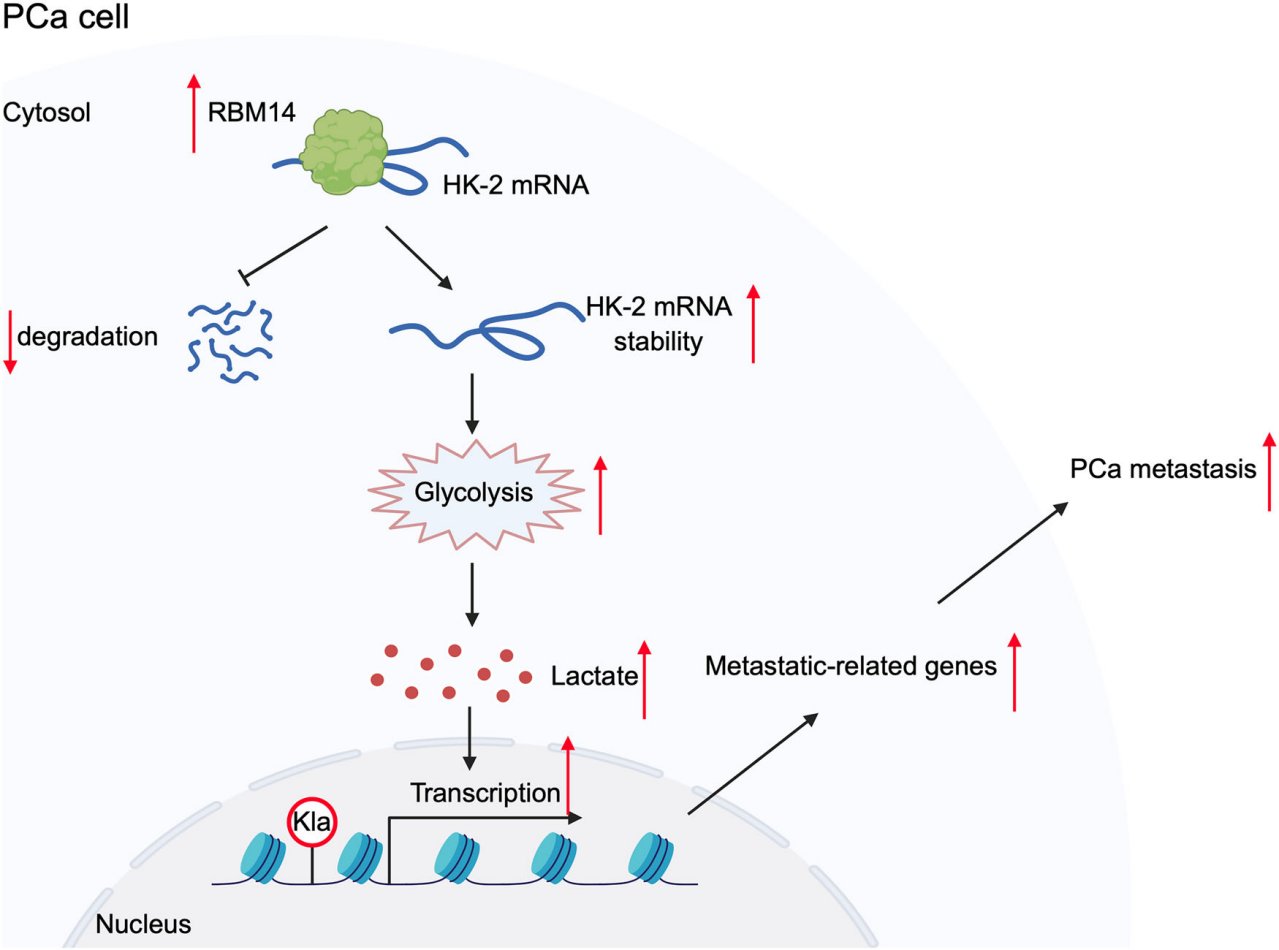
Schematic overview of the regulatory mechanism of RBM14 in renal cell carcinoma. This study reveals that RBM14 is markedly upregulated in aggressive prostate cancer. RBM14 facilitates PCa metastasis by stabilizing HK2 mRNA to enhance glycolysis, which elevates H3K18 lactylation and activates metastatic genes. Combined RBM14 inhibition and 2-DG treatment effectively restrains tumor metastasis.

In summary, our study suggests RBM14 as a novel driver of PCa metastasis through the RBM14/HK2/glycolysis/H3K18la axis. These findings also suggest that co-targeting RBM14 and glycolysis with 2-DG may represent a promising strategy for treating metastatic PCa (Fig. 8).

## Methods

### Clinical samples

Human PCa tissues were collected from patients in The Second Affiliated Hospital of Fujian Medical University (Quanzhou, China) from 2018 to 2022, following the ethics standards of the Helsinki Declaration. Informed consent was secured from all participants. The Ethical Committee have approved this study (Approval number: 2025-017).

### Cell culture and transfection

Cell lines 22RV1 and PC-3 were obtained from the Cell Bank of the Chinese Academy of Sciences (Shanghai, China). PC-3M were constructed by three rounds of metastasis screening using Transwell chambers. 22RV1 cells were cultured in RPMI-1640 medium (Gibco, USA). PC-3 and PC-3M cells were cultured in MEM medium (Gibco, USA). All cells were placed at 37 °C with 5% CO_2_.

### Construction of PC-3M cells

PC-3 cells were seeded into the upper compartment of Matrigel-coated Transwell chambers. After 24 h, the migrated cells adhering to the lower membrane surface of the chambers were detached with trypsin and cultured to expand the cell population to a sufficient quantity for the subsequent round of invasion selection. The expanded cells were then collected and re-seeded into the upper compartment of fresh Matrigel-coated Transwell chambers. This procedure was repeated three times to obtain PC-3M cells.

### Wound healing assays

The PCa cells were seeded in the 6 well plate. Until the cell confluence reached 90%, 200 μL pipette tip was used to scratch a straight. After washed by PBS for 3 times, the serum-free medium was added. The images were photographed at 0 h and 48 h. The wound healing area was measured using formula: wound healing area (%) = [(Initial wound area – 48 h wound area) / Initial wound area] × 100%

### Transwell migration assays

For transwell migration assays, the PCa cells suspended in serum-free medium and seeded into the upper compartment of transwell chamber (Corning, USA). The lower chamber was filled with complete medium. After 24 hours, the migrated cells were fixed and stained with 0.3% crystal violet for 15 min.

### Animal model

The experimental protocols were approved by the Ethics Committee of the Second Affiliated Hospital of Fujian Medical University (Approval number: 2025-017) and conducted in accordance with ARRIVE guidelines. For experimental metastasis assays, 4-week-old BALB/c nude mice were purchased from Shanghai SLAC Laboratory Animal Co., Ltd. (Shanghai, China). Luciferase-labeled PC-3 cells (PC-3-Luc or PC-3-Luc-oeRBM14, *n* = 5) at a density of 2 × 10⁵ cells were resuspended in 100 μl PBS and intracardially injected into the left ventricle. In vivo bioluminescent imaging was performed at 1 hour and 5 weeks post-injection to monitor cell distribution and metastasis.

For the RBM14 knockdown combined with 2-DG treatment experiment, mice were inoculated with PC-3-Luc or PC-3-Luc-shRBM14 cells and then randomized into 4 groups (*n* = 5). The mice were administered either saline or 2-DG (0.4%, w/v) via oral gavage daily for 5 weeks. After 5 weeks, in vivo bioluminescent imaging was conducted, following which the mice were euthanized humanely.

### Quantitative real-time PCR assays

Total RNA from cells and tissues was extracted using TRIzol reagent (Invitrogen, USA). The mRNAs were reversed transcribed using reverse transcription kit (Takara, Japan). Then, qPCR was used to detect the specific mRNA level using SYBR Green PCR master mix (Takara, Japan). The relative mRNA level was determined using 2^^-ΔΔCt^ method, and GAPDH used as internal control. The detailed primer sequences were listed in Supplementary Table S1.

### Western blot assay

For WB assay, cells and tissues samples were lysed using RIPA reagent (Beyotime Biotechnology, China). Protein samples were separated by SDS-PAGE and then transferred to the PVDF membranes (Bio-Rad, USA). The membranes were blocked by 5% BSA for 1 h at room temperature. Then, the membrane was incubated with the specific primary antibodies overnight at 4 °C. The next day, the membranes were incubated with the secondary antibodies followed by chemiluminescence. The detailed information of primary antibodies was listed in the Supplementary Table S2.

### RNA sequencing

RNA sequencing was performed by Novogene (Beijing, China). Sample quality control refers to the QC report. For transcriptome library prep, non-strand specific libraries purify mRNA via poly-T magnetic beads, synthesize cDNA (random hexamers), and go through end repair, adapter ligation, etc., quantified by Qubit/real-time PCR. Strand specific libraries use dUTP for second-strand cDNA and USER enzyme digestion. After library QC, pooling and Illumina sequencing (Sequencing by Synthesis) capture fluorescence signals for sequence data. For bioinformatics analysis: Fastp processes raw fastq data to get clean reads and calculates clean data’s Q20, Q30, and GC content; downstream analyses use high-quality clean data. HISAT2 (2.2.1) builds the reference genome index and aligns paired-end clean reads to it, using gene model annotations for splice-aware alignments. FeatureCounts (2.0.6) counts reads mapped to each gene, and FPKM is calculated to estimate gene expression. For differential analysis, DESeq2 (1.42.0, with replicates) is used, with *P*-values adjusted via Benjamini and Hochberg’s method (threshold: *p*adj ≤ 0.05 & |log2(foldchange)| ≥1).

### RNA Immunoprecipitation (RIP)

The RIP assays were performed using the Magna RIP Kit (Millipore) according to the manufacturer’s guidelines. Brieffly, 2 × 10^7^ PCa cells were used for RIP assays. After lysed, the lysis was incubated with RBM14 or Flag antibodies and corresponding beads. After washed for 5 times, the immunoprecipitated RNA was extracted and detected using qRT-PCR.

### RNA pulldown assay

Biotin-labeled HK2 mRNA probes (targeting the 4369–4594 region) were synthesized by GenePharma Technology (Shanghai, China). A non-targeting biotin-labeled scrambled probe was used as a negative control. Briefly, 5 × 10⁶ PCa cells were harvested and lysed on ice in lysis buffer supplemented with both protease inhibitors and RNase inhibitors. Then, the cell lysate was incubated with biotin-labeled HK2 probe or scrambled probe overnight at 4 °C. The beads were then washed five times with ice-cold wash buffer. The bound proteins were eluted by boiling in SDS-PAGE loading buffer and subjected to western blot analysis to detected by western blot assay.

### Extracellular acidification rate (ECAR) assays

The extracellular acidification rate was determined by ECARs using the Seahorse XF96 extracellular flux analyzer (Seahorse Bioscience) according to the manufacturer’s guidelines.

### Detection of lactate, ATP, and NADH/NAD+

Lactate levels were measured using the Lactate Detection Kit (Beyotime Biotechnology, China) following the manufacturer’s protocols. Briefly, 2 × 10⁵ PCa cells were lysed on ice, and the lysate was centrifuged to collect the supernatant. The kit-provided WST-8 working solution was mixed with the supernatant, and the mixture was incubated in the dark for 30 min. Lactate levels were then quantified by measuring absorbance at 450 nm using a microplate reader.

ATP levels were determined using the ATP Detection Kit (Beyotime Biotechnology, China) according to the manufacturer’s instructions. Approximately 2 × 10⁵ PCa cells were lysed on ice, followed by centrifugation to obtain the supernatant. The kit-provided ATP working solution was added to the supernatant, and after 5 s, ATP levels were measured using a luminometer.

NADH/NAD⁺ ratios were assessed using the NADH/NAD⁺ Detection Kit (Beyotime Biotechnology, China) following the manufacturer’s protocols. Briefly, 2 × 10⁵ PCa cells were lysed on ice, and the lysate was centrifuged to collect the supernatant. The kit-provided WST-8 working solution was mixed with the supernatant, and the mixture was incubated in the dark for 15 min. NADH and NAD⁺ levels were quantified by measuring absorbance at 450 nm using a microplate reader, and the NADH/NAD⁺ ratio was calculated accordingly.

### Chromatin immunoprecipitation (ChIP)-quantitative PCR

The ChIP assay was performed using the Simple ChIP Enzymatic Chromatin IP Kit (Cell Signaling Technology) following the manufacturer’s protocols. Briefly, PCa cells were crosslinked by 1% formaldehyde and incubating at room temperature for 10 min; the reaction was quenched with glycine for 5 min to terminate crosslinking. Cells were then harvested and the chromatin was sheared by sonication and followed by micrococcal nuclease digestion with micrococcal nuclease to gain the chromatin fragmentation. Then, H3K18la antibody or IgG was added to target specific chromatin overnight at 4 °C. Protein A/G agarose beads were then added, followed by incubation for 3 h at 4 °C. After washed by low-salt and high-salt solution. Proteinase K was used to digest protein-DNA complex. qPCR was performed to detected the specific DNA fragment using specific primers. The detailed primer sequences were listed in Supplementary Table S1.

### Statistical analysis

Each assay in this study was repeated at least three times. Data are expressed as mean ± standard deviation (SD). Statistical analyses were performed using GraphPad Prism 8.0 software. Differences between two groups were assessed using unpaired or paired Student’s *t* test, while differences among three or more groups were analyzed by one-way ANOVA. Correlations were evaluated using Pearson’s correlation test. *p* value < 0.05 was considered statistically significant.

## Supplementary information

Supplementary Figures

Original WB gel

Supplementary Tables

## Data Availability

The datasets used and/or analyzed during the current study are available from the corresponding author on reasonable request.
