# Supplementary material for: RBM14 drives prostate cancer metastasis via stabilizing HK2 mRNA to activate glycolysis and H3K18 lactylation
Source: Cell Death Discov. 2026 Apr 30;12:275. doi: 10.1038/s41420-026-03131-w (PMC13272929; doi:10.1038/s41420-026-03131-w)
Supplement: Supplementary file 1 — Supplementary Figures [file 41420_2026_3131_MOESM1_ESM.docx]

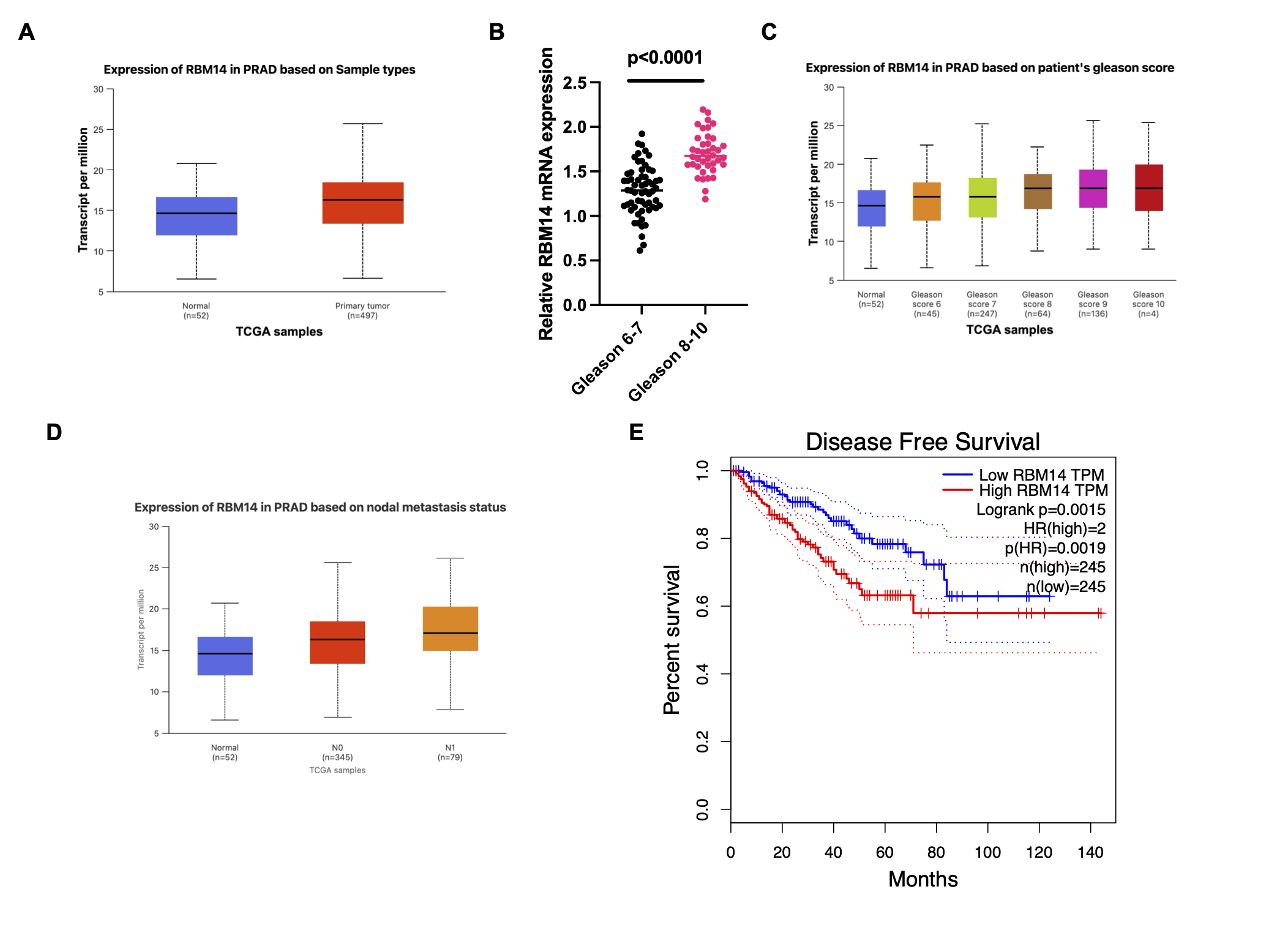


**Figure S1. RBM14 is upregulated in PCa tissues.** **A** TCGA-PRAD database analysis showing the RBM14 expression in PCa tissues and adjacent tissues. **B** RT-qPCR assays showing the RBM14 mRNA expression in Gleason 6-7 and 8-10 PCa tissues. **C** TCGA-PRAD database analysis showing the RBM14 mRNA expression in PCa patients with Gleason 6-7 or 8-10. **D** TCGA-PRAD database analysis showing the RBM14 mRNA expression in PCa patients with or without metastasis. **E** TCGA-PRAD database analysis showing the disease-free survival in PCa patients with high-RBM14 and low-RBM14 expression.


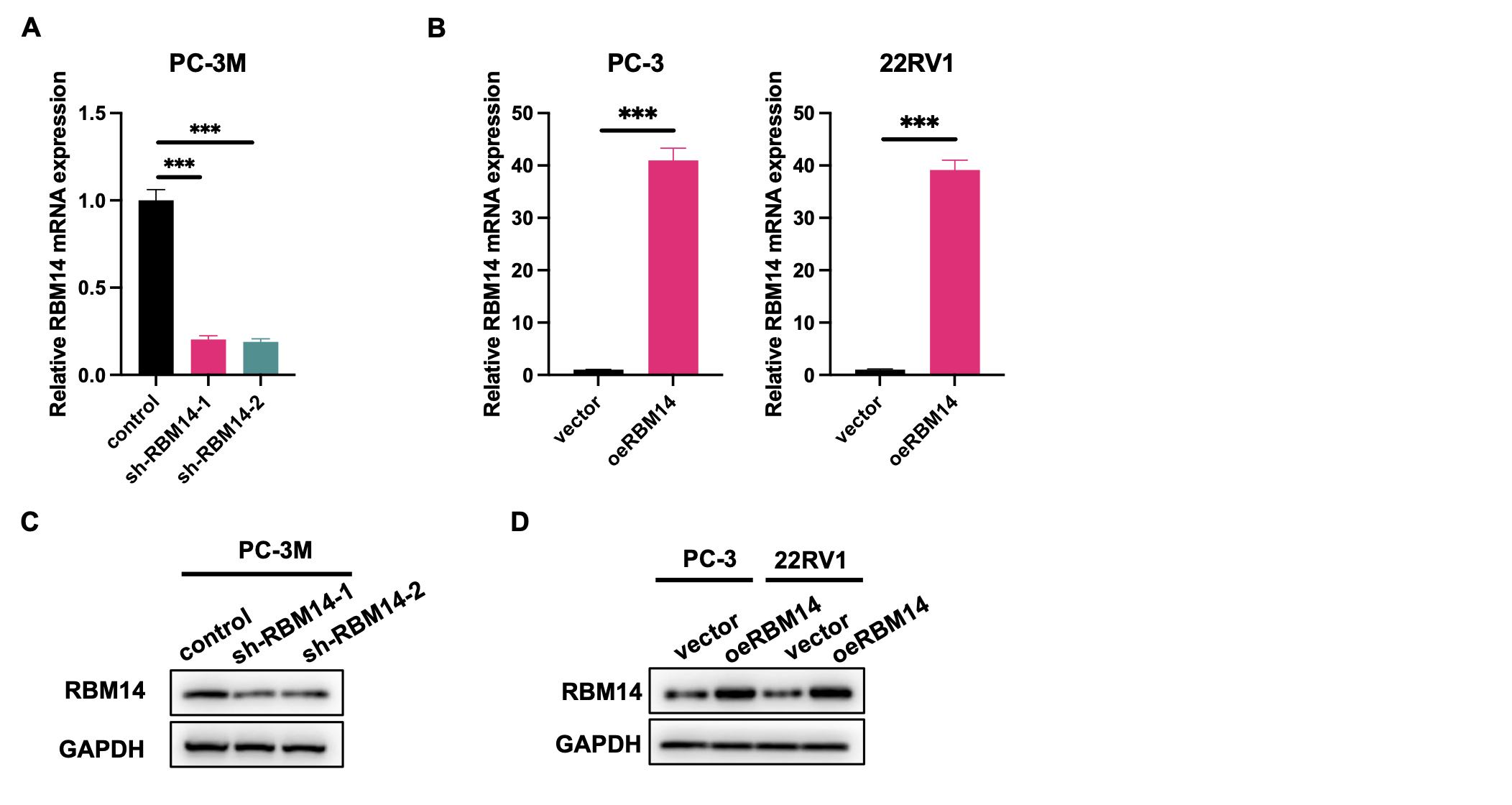


**Figure S2. Verification of RBM14 knockdown or overexpression efficiency.** **A** RT-qPCR assays showing the RBM14 mRNA knockdown efficiency in PC-3M cells. **B** RT-qPCR assays showing the RBM14 mRNA overexpression efficiency in PC-3 and 22RV1 cells. **C** Western blot assays showing the RBM14 mRNA knockdown efficiency in PC-3M cells. **D** Western blot assays showing the RBM14 mRNA overexpression efficiency in PC-3 and 22RV1 cells. Data were presented at mean±S.D. from at least three independent experiments. ***p < 0.001.


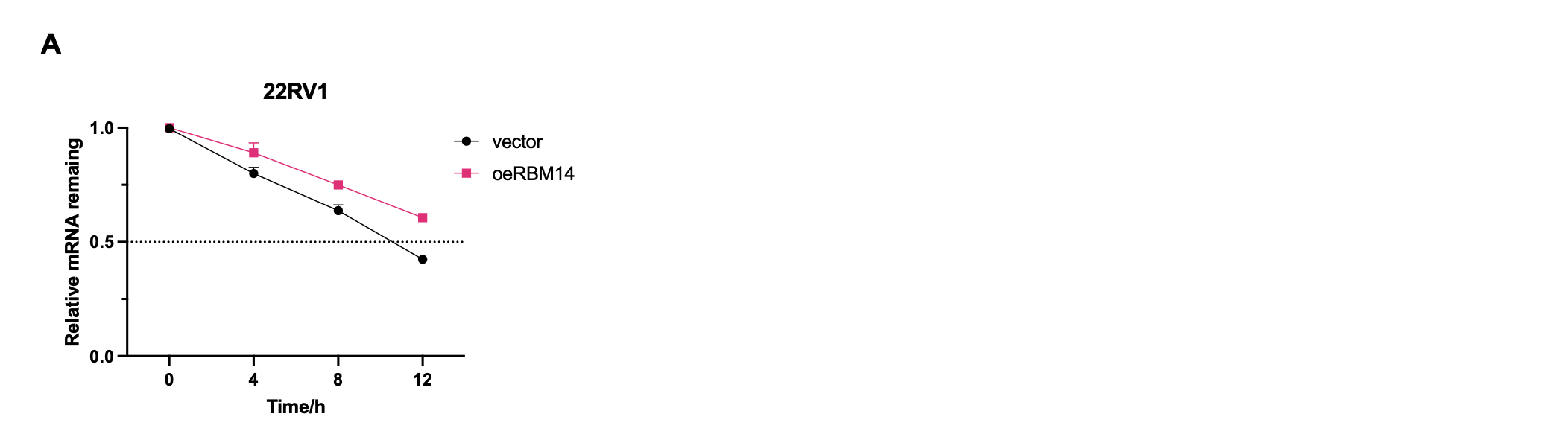


**Figure S3. RBM14 overexpression enhances HK2 mRNA stability. A** RNA stability assay showing the HK2 mRNA stability in 22RV1 cells with RBM14 overexpression or not. Data were presented at mean±S.D. from at least three independent experiments.


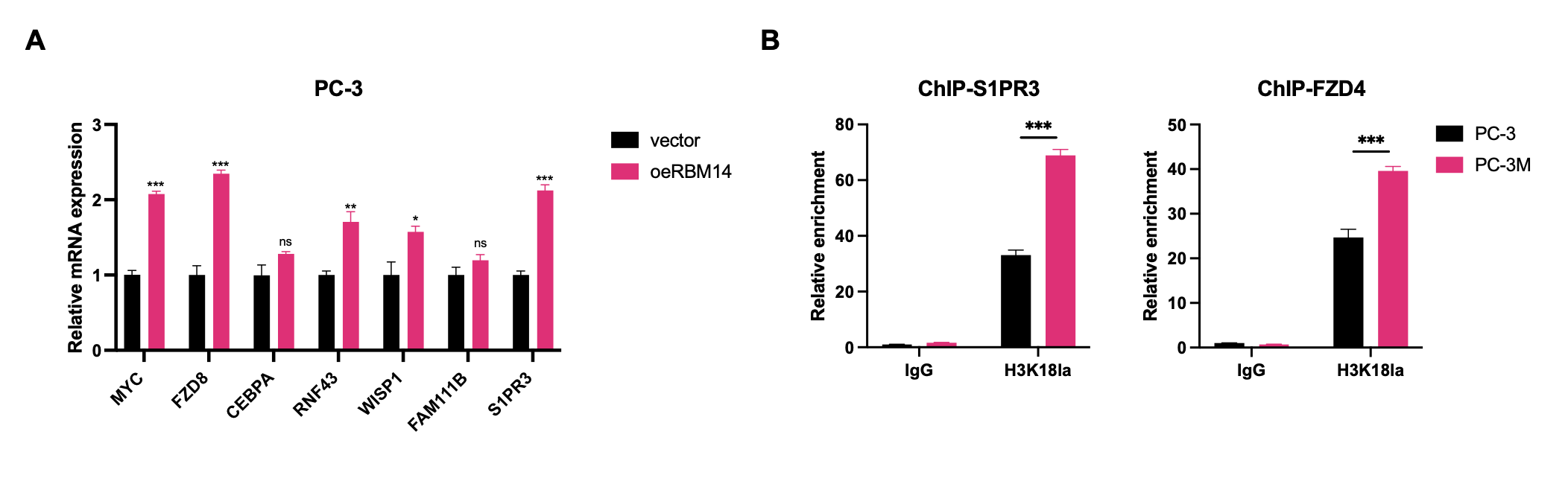


**Figure S4. RBM14 regulates metastasis-related gene expression. A** RT-qPCR showing the expression of MYC, RNF43, SIPR3, FZD8, CEBPA, WISP1, and FAM111B in PC-3 cells with RBM14 overexpression or not. **B** ChIP-qPCR assays showing the relative enrichment of H3K18la modification in the promoter region of SIPR3, FZD8 genes in PC-3 and PC-3M cells. Data were presented at mean±S.D. from at least three independent experiments. ns, not significant, *p < 0.05, **p < 0.01, ***p < 0.001.
