# Supplementary figures and images for: RBM14 drives prostate cancer metastasis via stabilizing HK2 mRNA to activate glycolysis and H3K18 lactylation
Source: Cell Death Discov. 2026 Apr 30;12:275. doi: 10.1038/s41420-026-03131-w (PMC13272929; doi:10.1038/s41420-026-03131-w)

Figure 1F

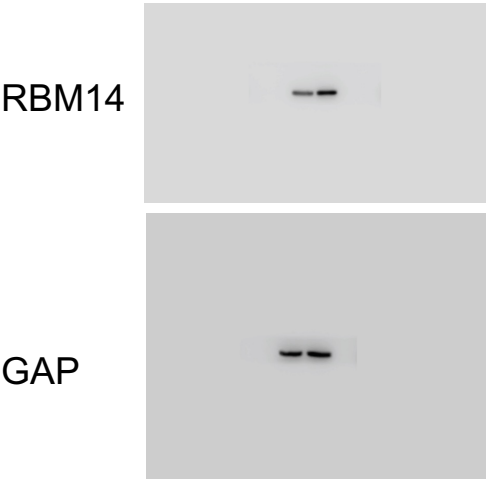

Figure 1H

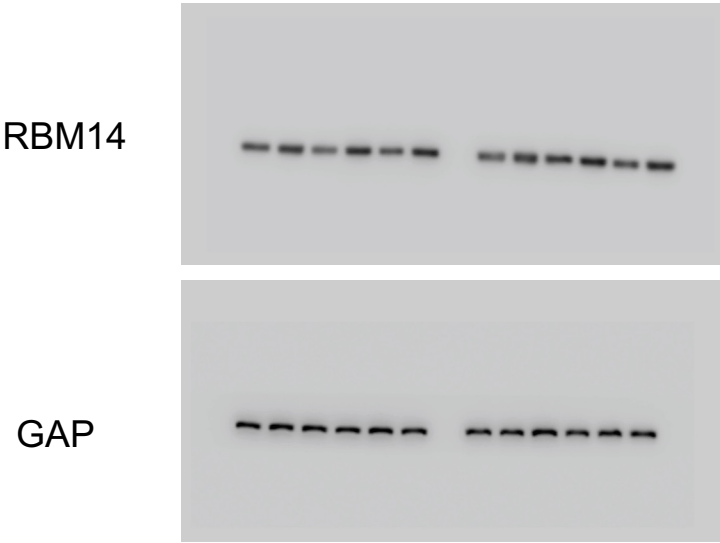

Figure 3E

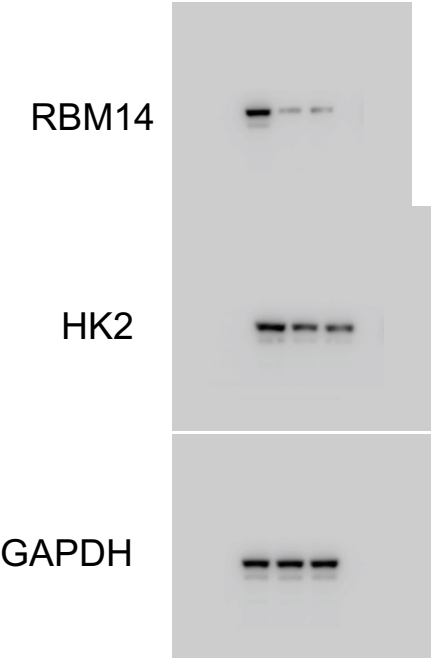

Figure 3F

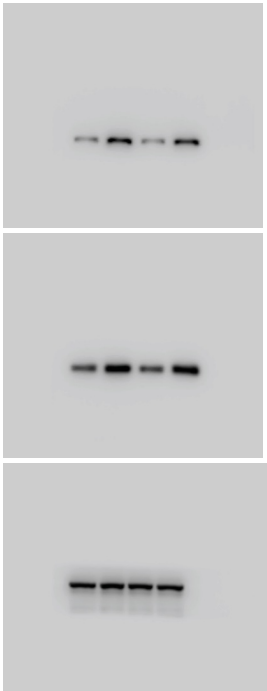

Figure 3H

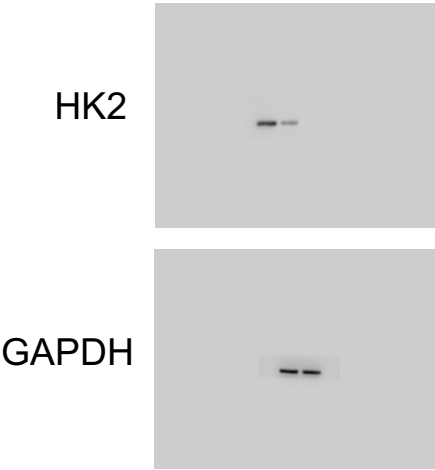

Figure 3L

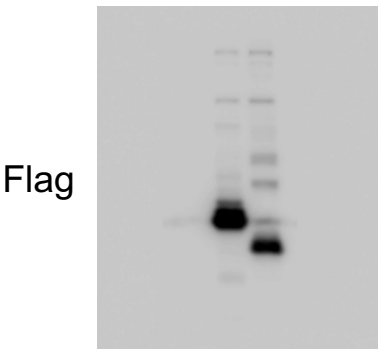

Figure 3O

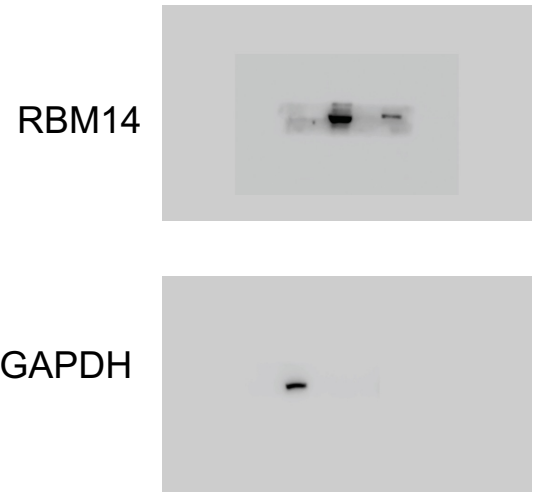

Figure 6A

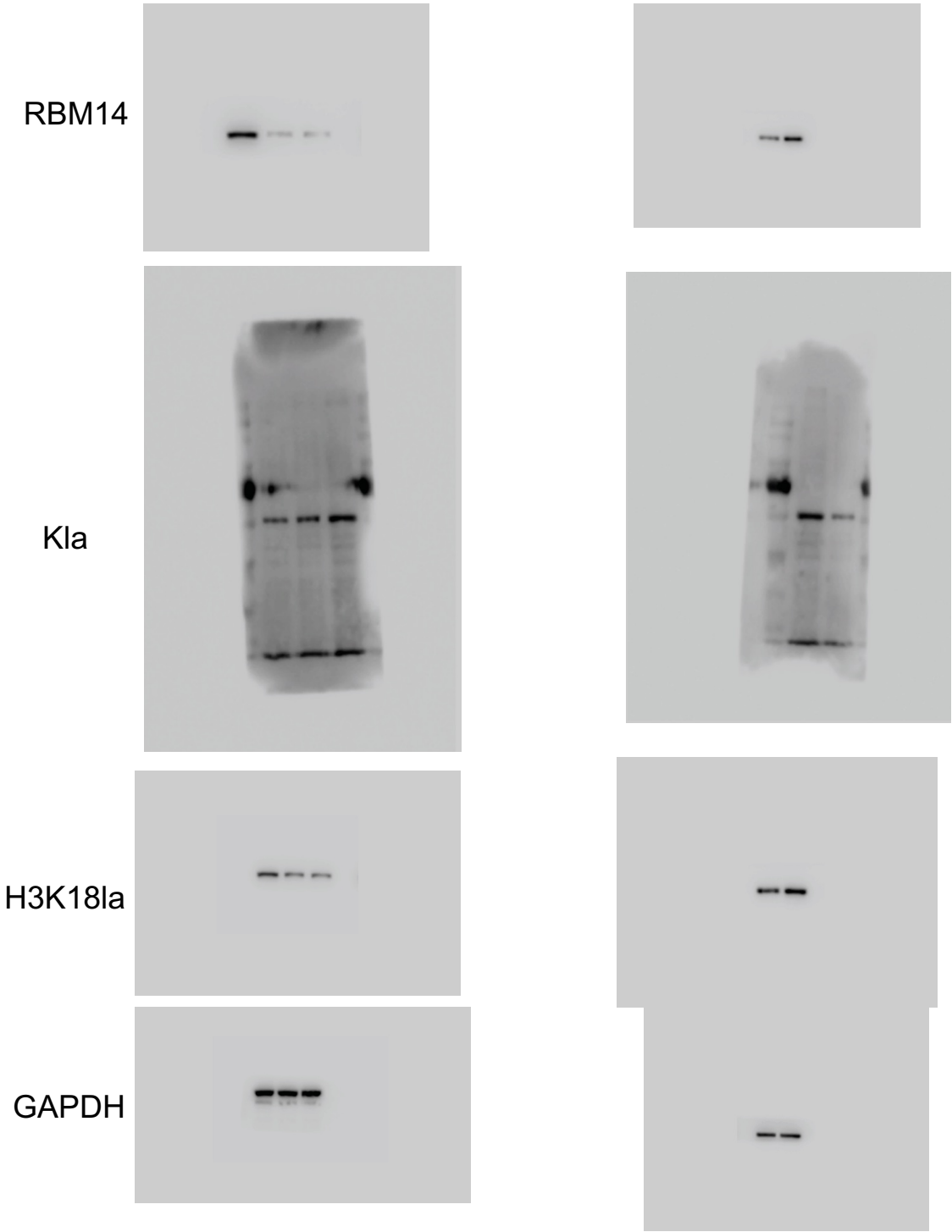

Figure S2

RBM14

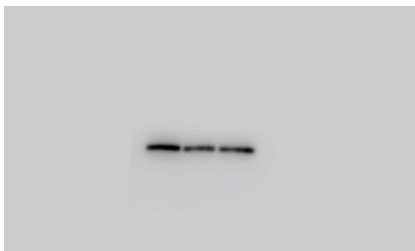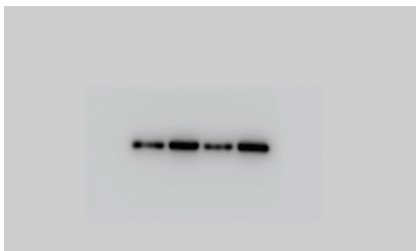

GAPDH

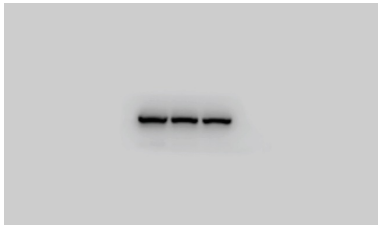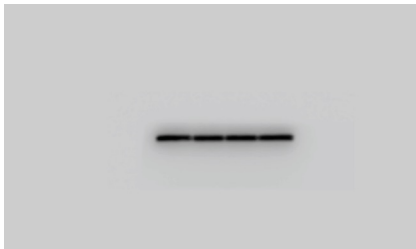

Supplement: Supplementary file 2 — Original WB gel [file 41420_2026_3131_MOESM2_ESM.pdf]
