# Supplementary material for: RBM14 drives prostate cancer metastasis via stabilizing HK2 mRNA to activate glycolysis and H3K18 lactylation
Source: Cell Death Discov. 2026 Apr 30;12:275. doi: 10.1038/s41420-026-03131-w (PMC13272929; doi:10.1038/s41420-026-03131-w)
Supplement: Supplementary file 3 — Supplementary Tables [file 41420_2026_3131_MOESM3_ESM.docx]

**Supplementary table S1**. The primer sequences used for RT-PCR.

| Gene Names | Sequences (5’-3’) |
| --- | --- |
| RBM14-F | ACTTGGAAGATTTTCGTGGGC |
| RBM14-R | CGGAACCATACCCTTGGTGG |
| HK2-F | GAGCCACCACTCACCCTACT |
| HK2-R | CCAGGCATTCGGCAATGTG |
| CCNL1-F | TACCATCGACCACTCTCTGATT |
| CCNL1-R | GGATGCGTAAGTCCGTCTCAC |
| DUSP5-F | GCCAGCTTATGACCAGGGTG |
| DUSP5-R | GTCCGTCGGGAGACATTCAG |
| ATF3-F | CCTCTGCGCTGGAATCAGTC |
| ATF3-R | TTCTTTCTCGTCGCCTCTTTTT |
| DDX28-F | TGCGAAAGCTCTCGTCTAAGG |
| DDX28-R | CCTCCTGTAGTGCGTGCAG |
| LCMT2-F | GCTCATTCACCGAGGCTACTA |
| LCMT2-R | CGCGGTTTTTAAGCGAAAATAGA |
| SOX21-F | GCTCGCCAATCCCGAGAAG |
| SOX21-R | ATCTCTGCCATTTTGGAGCCC |
| MMP9-F | AGACCTGGGCAGATTCCAAAC |
| MMP9-R | CGGCAAGTCTTCCGAGTAGT |
| MYC-F | GGCTCCTGGCAAAAGGTCA |
| MYC-R | CTGCGTAGTTGTGCTGATGT |
| RNF43-F | CATCAGCATCGTCAAGCTGGA |
| RNF43-R | TTACCCCAGATCAACACCACT |
| S1PR3-F | CGGCATCGCTTACAAGGTCAA |
| S1PR3-R | GCCACGAACATACTGCCCT |
| CEBPA-F | TTCACATTGCACAAGGCACT |
| CEBPA-R | GAGGGACCGGAGTTATGACA |
| WISP1-F | GTGCTGTAAGATGTGCGCTCA |
| WISP1-R | CCCGCTGTAGTCACAGTAGAG |
| FZD8-F | ATCGGCTACAACTACACCTACA |
| FZD8-R | GTACATGCTGCACAGGAAGAA |
| FAM111B-F | GCCCTTGAAATGCAGAATCCA |
| FAM111B-R | GCTGTAAACACACTACGGTCTAA |
| GAPDH-F | GGAGCGAGATCCCTCCAAAAT |
| GAPDH-R | GGCTGTTGTCATACTTCTCATGG |
| MYC-ChIP-F | ATAATGCGAGGGTCTGGACG |
| MYC-ChIP-R | TCTGAGAAGCCCTGCCCTT |
| RNF43-ChIP-F | AGAGCGAAAGAACGGGTCTG |
| RNF43-ChIP-R | CTGCTAGAGGCAACCACTCC |
| S1PR3-ChIP-F | TGGTCAGGATCTGGACAACG |
| S1PR3-ChIP-R | GACAGCGAGGGTTTGTTTGG |
| FZD8-ChIP-F | GTCTCTGCGGTCAATCCCAA |
| FZD8-ChIP-R | CTTAAGGCAGTCCCCGAGTG |

**Supplementary table S2**. The detailed antibodies information.

| Antibodies | Cat. number | Company |
| --- | --- | --- |
| RBM14 | ab70636 | Abcam |
| HK2 | ab209847 | Abcam |
| Flag | ab205606 | Abcam |
| Anti-Lactyl Lysine Rabbit | PTM-1429 | PTM BIO |
| H3K18la | PTM-1427RM | PTM BIO |
| GAPDH | ab181602 | Abcam |
